# Effect of sleeve gastrectomy on postprandial lipoprotein metabolism in morbidly obese patients

**DOI:** 10.1186/1476-511X-12-82

**Published:** 2013-06-01

**Authors:** Elisa Waldmann, Thomas P Hüttl, Burkhard Göke, Reinhold Lang, Klaus G Parhofer

**Affiliations:** 1 Medical Department II - Grosshadern, Ludwig-Maximilians University Munich, Munich, Germany; 2 Department of Surgery- Grosshadern, Ludwig-Maximilians University Munich, Munich, Germany; 3 Surgical Clinic, Bogenhausen, Munich, Germany; 4 Current address: Department of Surgery, Kreisklinik Ottobeuren, Germany

**Keywords:** Postprandial lipids, Chylomicron, Chylomicron remnant, Dyslipidemia, VLDL-triglycerides

## Abstract

**Background:**

Obesity is associated with abnormal fasting and postprandial lipids, which may link obesity with atherosclerosis. We explored fasting and postprandial lipids in morbidly obese patients treated with sleeve gastrectomy and in control subjects.

**Methods:**

After fasting for 12 h 15 morbidly obese patients (BMI 51.4±6.5 kg/m^2^, 43.7±12.6 years) received a standardized oral fat load before and 3 months after bariatric surgery (sleeve gastrectomy). Controls (n=9, BMI 23.1±1.4 kg/m^2^) were studied once. Plasma was obtained fasting and then postprandially every 2 h for 8 h. Triglycerides (TG), chylomicron-TG (CM-TG), VLDL/chylomicron-remnant (VLDL/CR)-TG, cholesterol, LDL-cholesterol, VLDL/CR-cholesterol and HDL-cholesterol were isolated by ultracentrifugation at each time point. Postprandial values were expressed as area under the curve (AUC) and incremental area under the curve (iAUC). In addition, fasting glucose and insulin values and HOMA-IR-Index was measured (n=14).

**Results:**

Compared to controls morbidly obese patients had elevated TG and slightly altered postprandial lipids. Following surgery (weight loss 23.4 kg±6.2 kg; p<0.001) fasting TG (−19.1%; p=0.04), VLDL/CR-TG (−20.0%; p=0.05) decreased significantly, while fasting cholesterol, VLDL-, HDL- and LDL-cholesterol did not change. AUC and iAUC decreased significantly for VLDL/CR-TG (−20.4%, p=0.04 and −38.5%, p=0.04, respectively). Neither fasting nor postprandial changes correlated with the change in weight. In patients with preoperatively elevated TG (>150 mg/dl) a similar pattern was observed. Fasting insulin and HOMA were reduced significantly (−51.9%; p=0.004 and −47.9%; p=0.011).

**Conclusions:**

Three months after sleeve gastrectomy fasting and postprandial lipoprotein metabolism and glucose metabolism is improved in morbidly obese patients. The potential mechanisms may relate to decreased caloric intake but also to hormonal changes.

## Background

Obesity has reached epidemic proportions with 1.5 billion overweight adults in 2008, of which 500 million were obese. The World Health Organization (WHO) predicts that by 2015 this number will grow to 2.3 billion overweight and 700 million obese adults [1]. Obesity is defined as a body mass index (BMI) (weight in kg divided by height in meters squared) of greater than 30 kg/m^2^: It is associated with an increased risk for hypertension, dyslipidemia, type 2 diabetes, coronary heart disease, stroke, gallbladder disease, osteoarthritis, dementia, sleep apnoea and respiratory problems, and various cancers [2,3].

Overall mortality is increased in obese subjects [4], with coronary heart disease being the major factor for higher mortality [5,6]. Lipid changes such as elevated TGs, elevated LDL-cholesterol (LDL-c) and low HDL-cholesterol (HDL-c) are typically found in obese patients and predispose to atherosclerosis.

The National Cholesterol Education Program (NCEP) recommends that blood lipid profiles are examined after an 8–12 h fast [7]. Fasting lipids are well-established independent risk factors for cardiovascular disease. However, from a lipidological point of view most subjects including obese persons are in a postprandial state for most of the day. Especially TG levels and thus the concentration of TG rich lipoproteins can increase enormously postprandially to very high levels and it may take more than 12 hours to reach fasting levels again [8]. It is therefore not surprising that in addition to fasting TGs the concentration of postprandial TGs has also been associated with cardiovascular events [9,10] and an independent atherogenic potential has been observed [11-14]. Newer data also highlight the importance of non-fasting remnant cholesterol [15] as a predictor of cardio-vascular events. Taken together these data indicate that postprandial lipoprotein metabolism plays an important role in atherogenesis.

As first-line therapy for obesity guidelines recommend lifestyle modification including physical activity as well as dietary and behavioral changes. Pharmacotherapy is considered second - line therapy if the patient does not respond to primary treatment. Finally, surgical therapy (bariatric surgery) is a treatment option in patients with a history of long-standing obesity with a BMI of 40 kg/m^2^ or a BMI greater than 35 kg/m^2^ and co morbidities such as diabetes. Of all treatment options surgery induces the most dramatic long-term weight loss [16]. A number of different surgical techniques are available for bariatric surgery (gastric banding, sleeve gastrectomy, bypass surgeries). These techniques differ considerably with respect to induced weight change, metabolic effects, and complication rates [17].

Numerous studies have evaluated the effect of bariatric surgery on fasting lipids, overall showing beneficial changes such as lowering cholesterol, TGs and LDL-cholesterol and increasing HDL-cholesterol [18-20]. The effect of weight loss on postprandial lipids has been rarely evaluated.

However, this may be particularly interesting because bariatric surgery, especially sleeve gastrectomy, not only reduces weight but also alters gastric motility and leads to earlier satiety, partly by earlier stimulation of the vagal nerve due to a reduction of gastric volume. Furthermore, during sleeve gastrectomy the ghrelin producing area is partly removed and it is believed that this may directly affect metabolic parameters (resulting in the term “metabolic surgery”).

Thus, the aim of our study was to evaluate the effect of bariatric surgery on fasting and postprandial lipid levels in morbidly obese patients and to compare the effect to normolipidemic control subjects.

## Results

All subjects had significant weight loss 3 months after surgery (mean weight loss 23.4 kg±6.2 kg; p<0.001).

**Figure 1:**
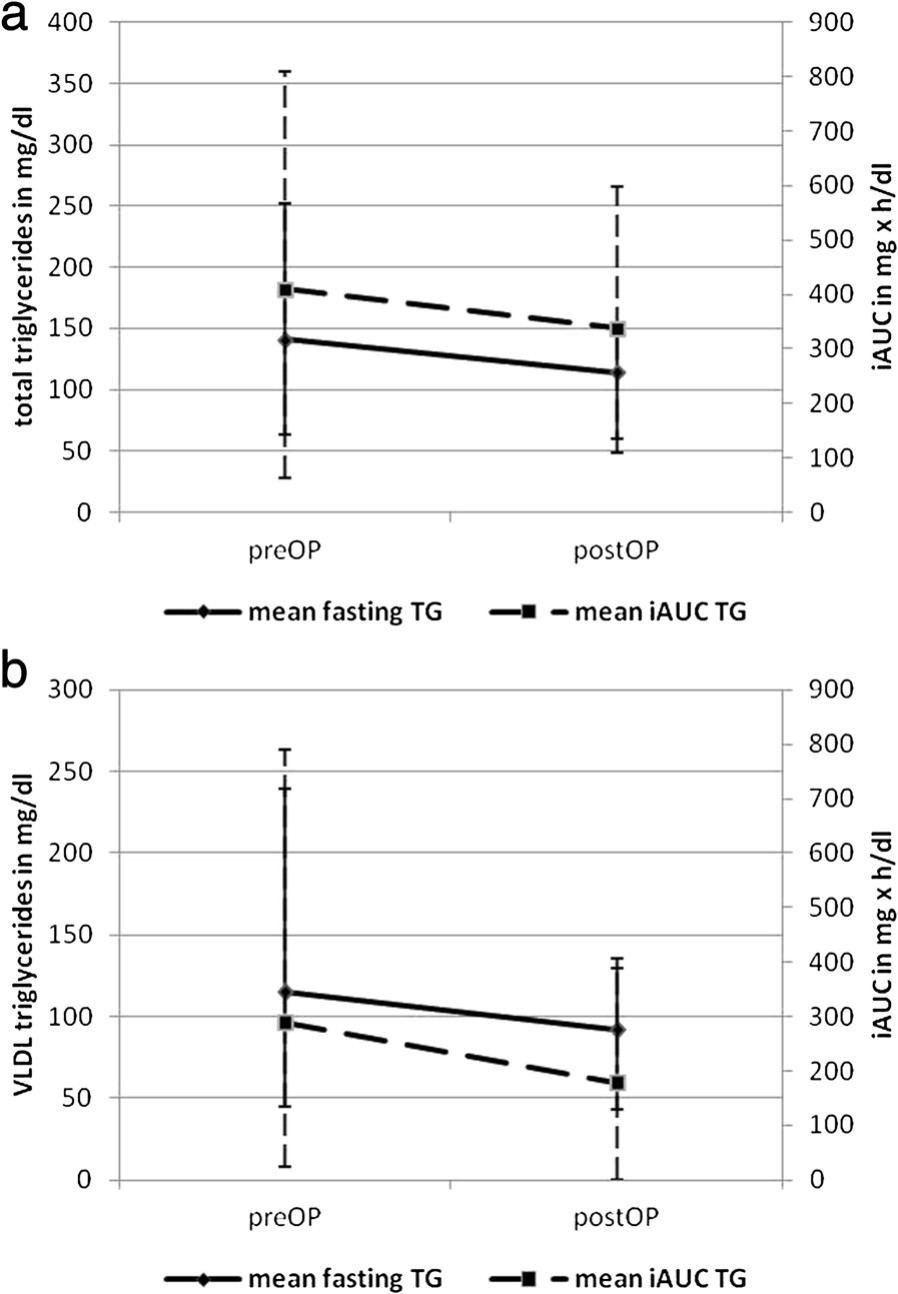
Mean values for fasting total (a) and VLDL (b) TGs and iAUC of total and VLDL TGs before and 3 months after sleeve gastrectomy (Mean + Maximum value/-Minimum value).

Fasting plasma (−19.1%; p=0.04) and VLDL/CR-TGs (−20.0%; p=0.05) decreased significantly (Figure 1) even beyond the level of the control group while plasma cholesterol and LDL-cholesterol were reduced and HDL-cholesterol was increased but did not change significantly (Table 1). Furthermore, there was a trend towards a decrease in the concentration of LDL/HDL-TGs (infranatant of ultracentrifugation).

**Table 1:** Fasting lipid parameters (mg/dl), AUC and incremental AUC (mg x h/dl) in patients before and after bariatric surgery and in control subjects (Data are expressed in mean ± SD); the indicated p-value relates to the comparison before-after surgery (as measured by Wilcoxon-test)

	**Controls**		**Before surgery**		**After surgery**		**Change [%]**	**p**
**Fasting**								
**Plasma-triglycerides**	117	±29	141	±42	114	±26	−19.1	**0.036**
**Plasma-cholesterol**	188	±42	175	±28	169	±31	−3.4	0.256
**HDL-cholesterol**	56	±14	42	±10	44	±9	+4.8	0.294
**LDL-cholesterol**	108	±35	105	±25	103	±29	−1.9	0.691
**VLDL/CR-triglycerides**	92	±28	115	±47	92	±27	−20.0	**0.053**
**VLDL/CR-cholesterol**	19	±11	20	±8	17	±5	−15.0	0.099
**LDL/HDL-triglycerides**	39	±11	39	±8	36	±7	−7.7	0.197
**AUC**								
**Plasma-triglycerides**	123	±412	1468	±490	1222	±309	−16.8	*0.078*
**CM-triglycerides**	377	±299	368	±214	288	±121	−21.7	0.670
**VLDL/CR-triglycerides**	789	±307	1078	±449	858	±295	−20.4	**0.036**
**iAUC**								
**Plasma-triglycerides**	396	±268	410	±238	338	±159	−17.6	0.394
**CM-triglycerides**	327	±254	306	±194	241	±129	−21.2	0.280
**VLDL/CR-triglycerides**	202	±126	288	±187	177	±140	−38.5	**0.044**

**Figure 2:**
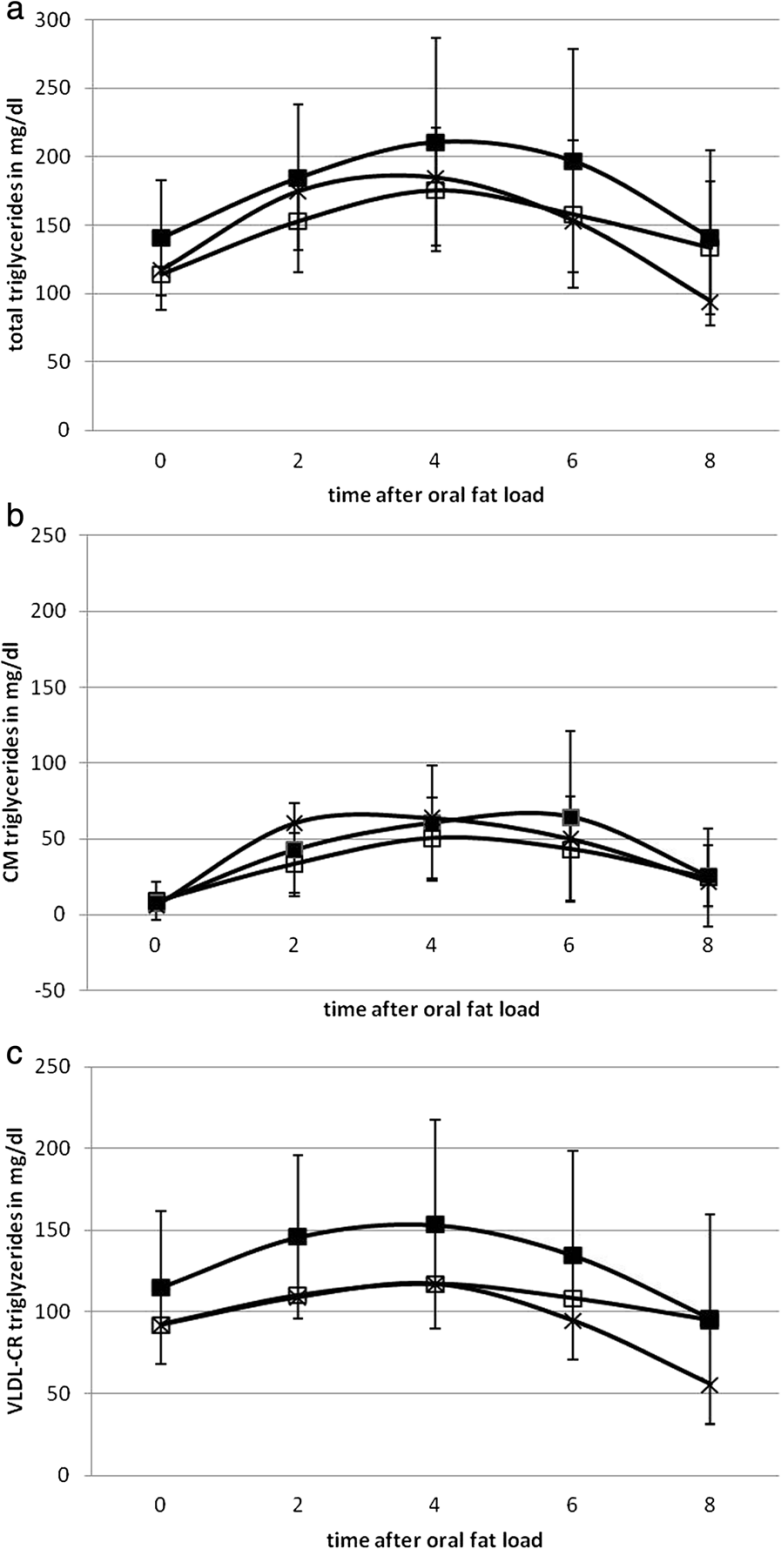
**Means (± SD) of plasma - (a), CM- (b) and VLDL/CR- (c) TGs following the oral fat load.** Filled and unfilled symbols represent data obtained before and after bariatric surgery. Crosses symbol control group.

Following the oral fat load, the area under the curve (AUC) for TGs decreased significantly for VLDL/CR-TG (−26.2%, p=0.017) (Table 1, Figure 2). The incremental area under the curve (iAUC) also changed significantly for VLDL/CR-TG (−17.6%; p=0.04) and improved for the other parameters, but these changes were not significant (Table 1). Furthermore, compared to controls, obese patients had a later peak of TG concentrations (plasma, CM and CR). Bariatric surgery induced a shift towards a later time point in the CR fraction while peak was shifted to an earlier time point in the CM fraction (Table 2, Figure 2). However none of these changes were significant.

**Table 2:** Time point (in hours) of peak TG concentration (mean ± SD) following an oral fat load; the indicated p-value relates to the comparison before-after surgery (as measured by Wilcoxon-test)

	**Controls**	**Before surgery**	**After surgery**	**Change [%]**	**p**
**Plasma-triglycerides**	2.89±1.45	3.87±1.41	4.53±1.77	17.24	0.42
**CM-triglycerides**	3.78±1.56	5.07±1.83	4.40±2.03	−13.16	0.36
**VLDL/CR-triglycerides**	3.11±1.76	3.87±1.60	4.27±2.25	10.34	0.52

Neither the changes in fasting values nor the postprandial changes correlated with the change in weight. We also did not observe any other meaningful correlations.

A subgroup analysis in patients with preoperatively elevated TGs (>150 mg/dl; n=5) indicated that patients with normal TG levels before surgery had neither significant changes in fasting data nor in AUC and iAUC data. In patients who had elevated fasting TG before the study a significant decrease of fasting plasma- and VLDL/CR-TG (−38%, p= 0.04; -45%, p=0.04) was observed. However, no significant changes in any of the postprandial parameters were seen (Table 3).

**Table 3:** Fasting lipid parameters and AUC (mg x h/dl; means ± SD) in patients with preoperatively high TGs (>150 mg/dl; n=5); the indicated p-value relates to the comparison before-after surgery (as measured by Wilcoxon-test)

	**Before surgery**		**After surgery**		**Change [%]**	**p**
**Fasting**						
**Plasma-triglycerides**	184	±36	114	±26	−38	**0.043**
**CM-triglycerides**	10	±5	9	±13	−10	ns
**VLDL/CR-triglycerides**	166	±37	92	±27	−45	**0.043**
**AUC**						
**Plasma-triglycerides**	1779	±597	1222	±309	−31	ns
**CM-triglycerides**	420	±229	288	±121	−31	ns
**VLDL/CR-triglycerides**	1400	±549	858	±295	−39	ns
**iAUC**						
**Plasma triglycerides**	432	±268	338	±159	−22	ns
**CM-triglycerides**	337	±205	241	±129	−29	ns
**VLDL/CR-triglycerides**	253	±253	177	±140	−30	ns

ns = not significant.

Analysis of parameters of glucose metabolism showed a significant reduction of fasting insulin (−51.9%, p=0.004), while glucose values did not change significantly. All but one patient (HOMA-IR = 2.22) had HOMA-IR >2.5. Insulin resistance improved significantly after surgery (−47.9%, p=0.01; Table 4). A subgroup analysis of patients with HOMA-IR >5 (n=9) compared to HOMA-IR <5 (n=5) showed a difference in absolute values of AUC and iAUC. However, due to the considerable inter-individual variation this difference was not statistically significant.

**Table 4:** Glucose (mg/dl), insulin-values (μU/ml) and HOMA-index in patients (n=14) before and after bariatric surgery (Data are expressed in mean ± SD); the indicated p-value relates to the comparison before-after surgery (as measured by Wilcoxon-test)

	**Before surgery**		**After surgery**		**Change [%]**	**p**
**Fasting**						
**Glucose**	100.1	±25.2	98.9	±23.9	−1.3	0.551
**Insulin**	28.9	±18.1	13.9	±5.9	−51.9	0.004
**HOMA**	6.8	±3.4	3.5	±2.1	−47.9	0.011

## Discussion

In this study we evaluated lipoprotein metabolism following bariatric surgery. Since it is well known that fasting levels of lipids improve considerably after bariatric surgery [18,19,21] we hypothesized that postprandial lipid metabolism would also significantly improve as shown by a reduction in the iAUC of TGs.

Three months after surgery fasting TGs as well as VLDL/CR-TGs decreased significantly by approximately 20% (Table 1). The decrease in lipid parameters is somewhat less than reported in previous studies [22,23]. In contrast to other studies we also did not see a significant change in HDL-cholesterol. The observed difference between ours and previous studies may relate to the fact that in previous studies reevaluation was performed 6 months or even 12 months after surgery, while our patients were studied three months after surgery and therefore had lost less weight. Although it would be most interesting to evaluate lipid metabolism before surgery, then at the time point of the most rapid weight loss and again at a new steady state, this is difficult to perform since the time course of weight loss is highly variable. In comparing our study to previous studies it should also be acknowledged that our subjects received a sleeve gastrectomy, a form of bariatric surgery for which only minimal data on lipid metabolism is available [20,24,25]. Thus, it could be hypothesized that the method of surgery has an impact on lipid levels. None of the other fasting lipid parameters changed significantly compared to baseline, although we observed a trend towards a higher HDL-cholesterol and lower total cholesterol.

In addition to the significant changes in fasting TGs, we also observed changes in postprandial lipoprotein metabolism. The observed alterations in AUC of VLDL/CR-TG primarily reflect the reduction of fasting concentrations. The primary endpoint, the more specific iAUC, also changed significantly for VLDL/CR-TG. This is in line with data on dietary induced weight reduction showing an improvement in fasting and postprandial lipids [26]. Similarly, it was shown that drugs for weight reduction lead to an improvement in fasting and postprandial TGs [27,28]. Although our results compare favorably to previously published data, some peculiarities of our study should be kept in mind: First, in our patients, the postoperative evaluation was performed while the subjects were still losing weight, thus before reaching a new steady-state, while studies evaluating diets or drugs were performed in steady-state. The already significant reduction in iAUC of VLDL/CR-TG 3 months after surgery indicates that further weight loss may result in a further improvement of postprandial lipoprotein metabolism. Second, since patients were not selected for abnormal lipids, they were characterized pre-operatively by a relatively normal fasting and postprandial lipoprotein metabolism, although obesity is associated with hypertriglyceridemia. There is no clear relationship between BMI and TGs. In fact, in our own experience less than half of patients presenting for bariatric surgery (n=52) have hypertriglyceridemia [20]. However, when we looked at the subgroup of patients with preoperatively elevated fasting TGs (>150 mg/dl) we did not observe a stronger effect of surgery on fasting or postprandial lipoprotein metabolism probably due to the small size of the group.

It is currently unclear which mechanisms are responsible for these changes. Most likely an improved handling of TG-rich lipoproteins (and not an altered production) is responsible for the improved postprandial lipoprotein metabolism. In particular, LPL activity and CETP mediated processes may change as a consequence of dramatic weight change or as a consequence of an improved glucose metabolism. Future studies need to address the underlying mechanisms.

Independent of the underlying mechanisms, the decrease in postprandial TG concentrations reflects a decreased number of remnant particles, which in the light of recent findings probably translates into a significantly decreased cardio-vascular risk.

Bariatric surgery induced a significant decrease in fasting insulin levels while fasting glucose did not change, resulting in a significant improvement of HOMA-IR index. This data is in line with previously published data [20,29], although it is especially interesting since previous studies showed same results after a longer period of follow up. Thus the positive effect on insulin sensitivity seems to be evident rather early after bariatric surgery. The reason for these changes may relate to the caloric restriction, the induced weight change or hormonal changes directly induced by bariatric surgery. Nevertheless these data are in good agreement with previous studies [30] showing that diabetes or insulin resistance may lead to an altered postprandial lipid response which in turn may contribute to the higher cardiovascular risk in diabetic patients.

A somewhat surprising finding of the study was that bariatric surgery had a heterogeneous effect on the peak time of TG concentrations in different lipoprotein fractions. While the peak shifted to an earlier time point in the CM fraction it shifted to a later time point in the VLDL/CR fraction. It should be noted that in the control subjects TG concentrations peaked earlier in all fractions. Since surgery induced a normalization of many metabolic parameters, one could have had speculated that peak time would decrease following bariatric surgery in all fractions. The reason for the observed delay in the VLDL/CR fraction is not known. A number of studies have indicated that bariatric surgery, especially sleeve gastrectomy, induces changes in the secretion and concentration of incretin hormones resulting in an altered gastro-intestinal motility [29].

Several limitations of our study should be noted. First the number of subjects evaluated in this trial is small, which raises the possibility of a type II error. Furthermore, the patient group was heterogeneous with respect to underlying lipid parameters. When we started our study it was unclear whether and to what extent patients undergoing bariatric surgery would benefit regarding their postprandial lipid metabolism. Subsequent studies should focus on more homogenous patient groups for example patients with well defined lipid disorders and should include a longer follow up period. Another limitation relates to the method used to evaluate postprandial lipid metabolism. We primarily relied on triglyceride concentrations. It is unclear whether the measurement of apoB-48 concentrations and/or the use of retinyl-palmitate as used in previous studies provide significant additional information [27,31]. Currently there is no widely accepted approach incorporating information from triglyceride, apoB-48 and retinyl palmitate into one model. However, future studies should incorporate further measurement of these parameters and in addition address aspects of potential underlying mechanisms.

## Conclusion

Three months after bariatric surgery fasting, postprandial lipoprotein metabolism and insulin sensitivity improved significantly in morbidly obese subjects. In the long-term this may lead to a reduction of cardiovascular events.

## Methods

Postprandial studies were performed in 15 morbidly obese subjects (6 male, 9 female, BMI 51.4±6.5 kg/m^2^, 43.7±12.6 years) and 9 normolipidemic controls (5 male, 4 female, age 34.0±10.5 years, BMI 23.1±1.4 kg/m^2^). Normolipidemic subjects were studied once and obese subjects were studied twice, once before bariatric surgery and once 3 months after surgery. All 15 obese subjects had bariatric surgery (sleeve gastrectomy) at the Department of Surgery – Grosshadern. Two of the study subjects were on simvastatin (10 mg/d) throughout the study period. The study was approved by the Ethics Committee of the Medical Faculty of the Ludwig-Maximilians-University Munich and all subjects gave written, informed consent for participation in the study.

### Postprandial study

The postprandial studies were performed as described previously [27] with slight modifications. Each postprandial study was performed after subjects had fasted for 12 h. After obtaining fasting blood, subjects received a fatty meal consisting of 50 g oil, 120 g cream, 20 g fluid egg, 10 g sugar, 1.8 g coffee flavor in a cream shake of 200 ml. Compared to previous studies the volume of the test meal had to be reduced to account for the reduced gastric volume of about 100 ml after bariatric surgery [32]. This fat load yields 858 kcal and contains 88 g fat, 188 mg cholesterol, 14 g carbohydrates, 5, 6 g proteins. It was ingested within 5 min. Following the fat load, blood samples were taken every 2 h for 8 h. During that time, subjects did not eat or drink calories but were allowed to drink water, tea and coffee without sugar or milk. This procedure was performed before and 3 months after bariatric surgery and once in normolipidemic controls.

### Analytical methods

Blood samples were drawn in tubes containing EDTA-sodium. Plasma was isolated by centrifugation (10 min, 3000 rpm). Ultracentrifugation of each plasma sample was performed to obtain two fractions of TG-rich lipoproteins: CM and VLDL/CR. To separate CM from VLDL/CR, 3 ml of plasma in a Beckman-tube were overlaid with a solution of d=1.006 kg/l and centrifuged for 20 min at 20,000 rpm (Ti 50.4 rotor; Beckman Coulter, Inc., Palo Alto, CA). The supernatant, containing the CMs, was removed using a Beckman tube slicer, whereas the infranatant was used to obtain VLDL/CR by further ultracentrifugation (d=1.006 kg/l, Ti 50.4 rotor, 18 h, 40,000 rpm). In plasma and in both lipoprotein fractions, cholesterol and TG concentrations were determined. In plasma the concentrations of HDL-cholesterol and LDL-cholesterol were also determined. TG and cholesterol concentrations were measured by using the Alcyon lipid analyzer (Alcyon 300; Abbott Laboratories, IL) and the appropriate commercial kit (Roche Molecular Biochemicals). Postprandial metabolism was quantified by calculating the area under the curve (AUC) and the incremental AUC for plasma-, CM- and VLDL-TGs and -cholesterol, plasma HDL, LDL, TGs and cholesterol in the infranantant. Concentrations obtained over the 8 h period following the ingestion of the fat meal were used for this calculation. Areas under the curve (AUC) for TGs were calculated using the linear trapezoidal method as previously described [27]. The incremental AUC was determined as the AUC minus the baseline or fasting concentration.

Fasting insulin and glucose were determined in plasma samples by the Department of Clinical Chemistry at the University of Munich (LMU) using photometry and elektrochemiluminescence (Beckmann Coulter AU680 and Roche Cobas E 411). Insulin sensitivity was evaluated using the Homeostasis Model Assessment of Insulin Resistance (HOMA-IR) as previously described [33].

### Statistical analysis

A paired comparison was performed to analyze our data. The primary endpoint was the incremental area under the TG curve following the standardized oral fat challenge. The sample size estimation (n=16) is based on a two-sided significance of 0.05, a statistical power of 80%, an estimated change of 10% and a variance of 15%. Initially 20 patients undergoing bariatric surgery, including sleeve gastrectomy (n=15), gastric banding (n=3) and gastric bypass (n=1) were recruited for the study. Due to the great heterogeneity related to the different surgical methods only patients undergoing sleeve gastrectomy were chosen for this analysis. Differences between parameters obtained before and after bariatric surgery were evaluated by calculating arithmetic means ±SD. Range is given in parenthesis. To test for statistical significance we used non parametrical Wilcoxon test analysis. To measure linear relationship we used Pearsons Correlation coefficient. These tests were performed using the SPSS, Inc. software (SPSS, Inc., Chicago, IL). The critical P value for significance was set at 0.05.

## Competing interests

E. Waldmann, T. Hüttl, B. Göke, R. Lang, and K. Parhofer have no conflict of interest. There were no benefits in any form from a commercial party related directly or indirectly to the subject of this manuscript or the authors.

## Authors’ contributions

EW: Assessment and analysis of source data, corresponding author. KGP: Design of the study, supervision of study, analysis of data, revision of article. TPH: Responsible surgeon, recruitment of patients, revision of the article. RL: Responsible surgeon, recruitment of patients, revision of the article. BG: Revision of the article. All authors read and approved the final manuscript.
